# Quantifying bacterial attachment and detachment using leaching solutions of various ionic strengths after bacterial pulse

**DOI:** 10.1186/s13568-017-0340-2

**Published:** 2017-02-14

**Authors:** Nag-Choul Choi, Jae-Woo Choi, Kyu-Sang Kwon, Sang-Gil Lee, Soonjae Lee

**Affiliations:** 1 Department of Rural Systems Engineering/Research Institute of Agriculture and Life Sciences, Seoul National University, Seoul, 08826 Republic of Korea; 2 Center for Water Resources Cycle Research, Korea Institute of Science and Technology, Hwarang-ro 14-gil 5, Seongbuk-gu, Seoul, 02792 Republic of Korea; 3 Department of Earth and Environmental Sciences, Korea University, Anam Dong 5-1, Sungbuk-gu, Seoul, 02841 Republic of Korea

**Keywords:** Bacteria transport, Attachment, Reversibility, Ionic strength, Chemical perturbation, DLVO

## Abstract

In this study, we quantified the attachment and detachment of bacteria during transport in order to elucidate the contributions of reversible attachment on bacterial breakthrough curves. The first set of breakthrough experiment was performed for a laboratory sand column using leaching solutions of deionized water and mineral salt medium (MSM) of 200 mM with reference to KCl solution by employing *Pseudomonas putida* as a model bacterium. In the second set of experiment, the ionic strengths of leaching solutions immediately after bacterial pulse were lowered to tenfold and 100-fold diluted system (2 and 20 mM MSM) to focus on the influence of physicochemical factor. Results have shown that bacterial retention occurred in the sand column due to the physical deposition and physicochemical attachment. The physicochemical attachment was attributed to the high ionic strength (200 mM MSM) of leaching solution and the formation of primary energy minimum. Replacing the 200 mM leaching solution with the lower ionic strengths after pulse resulted in the increased tailing of breakthrough curve due to the detachment from the attached bacteria. The detachment could be well explained by DLVO theory, which showed the formation of energy barrier and disappearance of the secondary minimum as the ionic strength gradually decreased. Analysis of mass recovery revealed that 12–20% of the attachment was due to physical and physicochemical attachment, respectively, where the latter consisted of 25–75% of irreversible and reversible attachment respectively.

**Electronic supplementary material:**

The online version of this article (doi:10.1186/s13568-017-0340-2) contains supplementary material, which is available to authorized users.

## Introduction

Transport of bacteria through porous medium has been an important issue for the purpose of bioaugmentation scheme as well as for the prediction of movement of pathogenic microorganisms in aquifer systems. Aquifer systems generally exhibit a low nutrient condition and therefore injection of nutrients medium is often required during application of contaminant-degrading bacteria in order to sustain the bacterial population. It was found that nutrient limitation affected substantially the transport of bacteria through sand (Priestley et al. 2006). In the study of bacteria transport (Choi et al. 2007), mineral salt medium (MSM) with a rather high ionic strength was used to maintain the cell density of the injected bacterial solution during transport.

Numerous studies have shown that bacteria transport are dependent on ionic strength of leaching solutions, and that higher ionic strength led to higher irreversible attachment (Mills et al. 1994), collision (Abramson and Brown 2007; Jewett et al. 1995; Li and Logan 1999) or sticking efficiency (Bolster et al. 2001), and lower mass recovery (Choi et al. 2007; Gannon et al. 1991; Kim et al. 2009a) or deposition rate (Chen and Zhu 2004; Chen and Walker 2007; Kim et al. 2009a, b; Kuznar and Elimelech 2004; Redman et al. 2004; Rijnaarts et al. 1996), all of which may result in an unfavorable condition for bioaugmentation scheme. The occurrence of effluent with lower cell density when leaching solutions with high ionic strength were used is attributed to the formation of the primary energy minimum near the particle surface as a result of compressed electrical double layer, and thereby domination of the London-van der Waals attractive force over the bacteria-surface interaction energies at all separation distances.

One of the methods to overcome the bacterial loss was to apply leaching solution with low ionic strength in the column tests (Gannon et al. 1991; Redman et al. 2004). They found that the decrease in the ionic strength of the pore fluid—thereby eliminating the secondary energy minimum—resulted in the release of the majority of previously deposited bacteria. However, their findings were limited to the investigation on the existence of the secondary energy minimum since they applied the leaching solution with low ionic strength after a complete bacterial breakthrough—a rather long elution after breakthrough—in the column tests. More detailed information on the separation of the primary energy minimum from the secondary energy minimum and the reversible portion of bacterial attachment requires a careful design of breakthrough experiment for a given bacterium and porous medium. One of the solutions to this problem would be to apply leaching solutions with various ionic strengths immediately after a bacterial pulse since the input of lower ionic strength will generate various situations in the interaction energy between bacteria and particle surfaces as leaching solutions propagate down the column system. The objective of this study is to investigate the reversibility of bacterial attachment during the transport through aquifer material and the effect of the chemical perturbation on the bacterial detachment. We conducted bacterial transport experiments by applying leaching solutions of various ionic strengths after bacterial pulse. The bacterial attachment and detachment were quantified through comparison of mass recoveries of bacterial breakthrough curves.

## Materials and methods

### Organisms and culture preparation

Benzene-degrading bacteria, *Pseudomonas putida* KCTC-1769, was obtained from Korean Center for Type Cultures, Seoul, South Korea. Bacterial culture was prepared as described previously (Kim et al. 2005). Initially, the bacteria in a freeze-dried state were revived in 250 ml Erlenmeyer flasks containing 100 ml of LB medium over a period of 2 days. One milliliter of culture was transferred to a volume of 500 ml LB broth and incubated at a 30 °C temperature in a 140 rpm orbital shaker. Cells were harvested in the late exponential growth phase by centrifugation.

For column experiments, the cells were washed two times and suspended in deionized water or mineral salt medium (MSM) with pH of 7 and with ionic strength of 200 mM containing following constituents per liter of distilled water: K_2_HPO_4_, 6 g; KH_2_PO_4_, 4 g; (NH_4_)_2_SO_4_, 2 g; MgCl_2_, 6.6 g; CaCl_2_, 2.5 g; ZnSO_4_, 0.8 g; NiSO4, 0.24 g; (NH_4_)_6_Mo_7_O_24_, 0.18 g; CuSO_4_, 0.03 g; MnSO_4_, 0.5 g; CoCl_2_, 0.19 g; FeCl_2_, 0.06 g (Kim et al. 2005), and adjusted to an optical density of 0.5 at 600 nm (OD_600_). For the surface potential measurement, cells were washed and suspended with 200 mM MSM and 10-, 100-, 1000-fold diluted MSM. All glassware and materials were washed and sterilized in the autoclave at 121 °C for 15 min to prevent any influence by other microorganisms. Effective diameter and zeta potential of cell were determined by surface potential analysis.

### Porous media

Sand materials (Jumunjin silica, South Korea) were used for column tests. Prior to use, mechanical sieving was performed using US standard sieves (Fisher Scientific), No. 30 and 10 so that sand fractions (0.6 mm < ϕ < 2.0 mm) could be retained. After sieving, the quartz sands were washed using deionized water to remove any microcolloids which can interrupt the measurement of optical density of effluent samples, and then were autoclaved for 20 min in 121 lb pressure and oven-dried at 70 °C for 3–5 days. Previous study (Choi et al. 2007) revealed that the sand materials mainly consisted of quartz. The zeta potential of sand material were determined by surface potential analysis.

### Surface potential analysis

**Fig. 1:**
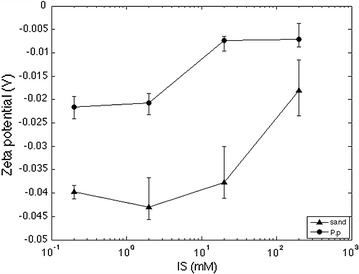
Zeta potential of *Pseudomonas putida* and quartz sand as a function of ionic strength

The Zeta potentials were measured at 25 °C using a ZetaPALS analyzer (Brookhaven Instruments Corporation, Holtsville, NY) and repeated five times for each suspension of ionic strength from 0.2, 2, 20, 200 mM. Zeta potentials of cell and quartz sand are presented in Fig. 1. Both bacterial cell and quartz sand were negatively charged over the range of ionic strength from 0.2 mM to 200 mM. The quartz sands exhibited more negative charge (−39.7 to −18.1 mV) than *P. putida* (−21.6 to −7.1 mV) (Table 1). The effective diameter of *P. putida* was determined using an automatic particle sizer (90Plus, Brookhaven Instruments Corporation). The effective diameter of the cell suspended in MSM (0.5 OD_600_) was 1516.3 nm.

**Table 1:** Electrophoretic mobility and zeta potential of *Pseudomonas putida* and quartz sand

IS (mM)	*P. putida*		Quartz sand	
	Mobility (um/s)/(V/cm)	Zeta potential (mV)	Mobility (um/s)/(V/cm)	Zeta potential (mV)
0.2	−1.69E+00	−2.16E+01	−3.10E+00	−3.97E+01
2	−1.62E+00	−2.07E+01	−3.35E+00	−4.30E+01
20	−5.77E−01	−7.38E+00	−2.95E+00	−3.77E+01
200	−5.55E−01	−7.10E+00	−1.42E+00	−1.81E+01

### Column experiments

**Fig. 2:**
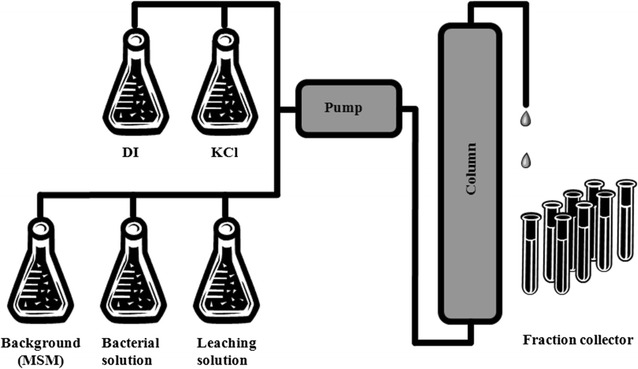
Schematic diagram of column experiments

A plexiglass column with a diameter of 25 mm and a height of 300 mm was packed with quartz sands, and the bulk density and porosity were found 1.65 g/cm^3^ and 0.37 respectively. The sand column was then saturated with deionized water by allowing upward flow to minimize air entrapment using a variable speed pump (Fluid Metering, Syosset, NY, USA) until steady-state flow condition was achieved (Fig. 2). Once a constant flow rate of 1.0 ml/min (pore water velocity of 0.55 cm/min) was established, the first set (Exp. A, B) of breakthrough experiment was performed by applying a pulse-type injection of 45 ml KCl (Exp. A) and bacterial solution (Exp. B) of 0.5 OD_600_ for 45 min (0.82 PV) in order to examine the physical factor affecting the bacteria transport. As soon as the KCl or bacterial pulse injection was completed, deionized water was again introduced under steady-state flow condition. The second set of the experiment (Exp. C_1_, C_2_, C_3_) was conducted by applying the same pulse conditions described in the first set, except for bacterial suspension in 200 mM MSM with leaching solutions of three different ionic strengths (200, 20, 2 mM) immediately after bacterial pulse, in order to examine the physicochemical factor which will influence the interaction energy-separation distance relationship. Details of experimental conditions are given in Table 2. Effluent samples were collected using a fraction collector (Model: RTRV II, Tucson, AZ, USA). The KCl concentration and ionic strength of the effluent were analyzed using the electrical conductivity meter (Orion, Model: 130A, Germany) while bacterial concentration was analyzed using Heyios ß UV spectrophotometer (Thermo-Electron Corporation). The mass recoveries (MR) of KCl and bacteria were quantified by the ratio of the eluted to injected KCl or cells as following

**Table 2:** Details of experimental conditions imposed on the column experiments

Exp.	Conditions for pulse input				IS (mM) of leaching solution			Type of leaching solution
	Q (ml/min)	t_0_ (min)	V_0_ (ml)	C_0_ (OD_600_)	Before^a^	During^b^	After^c^	
A	1.0	45	45	0.5	0	13.4	0	KCl
B	1.0	45	45	0.5	0	0	0	DI
C_1_	1.0	45	45	0.5	200	200	200	MSM
C_2_	1.0	45	45	0.5	200	200	20	MSM
C_3_	1.0	45	45	0.5	200	200	2	MSM

^a, b, c^ Before, during and after bacterial pulse respectively

1 $$MR = \frac{1}{{C_{0} t_{0} }}\mathop \int \limits_{0}^{\infty } C(t)dt$$

where *C*_0_ and *C*(*t*) are input and effluent concentrations respectively, and *t*_0_ (min) is the duration of tracer injection.

### Surface interaction energy

The interaction energy between the bacterium and the quartz sand surface was calculated by DLVO theory (Elimelech and Omelia 1990). Since bacteria and colloid particles show the similarity in size and surface charge, the bacterial deposition can be regarded to follow the principles of colloid chemistry. According to the DLVO theory, the total interaction energy (V_Tot_) can be expressed as the sum of electrostatic double layer (V_EDL_) and Van der Waals interaction energy (V_VdW_).

2 $$V_{Tot} = V_{EDL} + V_{VdW}$$

The electrostatic double layer interaction can be obtained as following (Hogg et al. 1966):

3 $$V_{EDL} = \pi \varepsilon_{0} \varepsilon_{r} a_{p} \left\{ {2\psi_{p} \psi_{c} \ln \left( {\frac{{1 + { \exp }( - \kappa h)}}{{1 - { \exp }( - \kappa h)}}} \right) + \left( {\psi_{p}^{2} + \psi_{c}^{2} } \right)\ln \left( {1 - { \exp }( - 2\kappa h)} \right)} \right\}$$

where ε_0_ is the vacuum permittivity (8.85e−12 C^2^/N/m^2^), ε_r_ is the relative dielectric permittivity of water (80.1), a_p_ is the equivalent spherical radius of the bacteria (m), h is the separation distance between the bacterium and the collector surface (m), ψ_p_ and ψ_c_ are the surface potentials of the bacterial cell and the collector surface (V). The inverse of Debye length (1/m), κ, is given by (Russel et al. 1989).

4 $$\kappa = \sqrt {\frac{{2e^{2} N_{A} I}}{{\varepsilon_{0} \varepsilon_{r} kT}}}$$

where e is the electron charge (1.602 × 10^−19^ C), N_A_ is Avogadro’s number (6.02 × 10^23^ molecules/mol), I is ionic strength (mol/l), k is Boltzman’s constant (1.381 × 10^−23^ J/K), and T is temperature (K). The van der Waals interaction potential was calculated using the following (Elimelech and Omelia 1990):

5 $$V_{VdW} = - \frac{{Aa_{p} }}{6h} \left( {1 + \frac{14h}{\lambda }} \right)^{ - 1}$$

where A is Hamaker constant for bacteria-water-surface collector (6.5 × 10^−21^ J for quartz), and λ is characteristic wavelength of the dielectric (m). To investigate the effect of ionic strength on the bacterial attachment and detachment, the interaction energy profiles under various ionic strength of 0.1–200 mM were calculated using the measured and interpolated zeta potentials.

The foregoing DLVO model could not describe the portion of the physical deposition, although the effect of ionic strength on bacterial transport could be described appropriately. Recent studies suggested that non-DLVO interactions are related to the bacterial deposition in aqueous system including interactions such as hydrophobic interaction, hydration pressure, structural forces, steric interaction and Born repulsion (Redman et al. 2004; Rijnaarts et al. 1999; Wang et al. 2011). Based on extended DLVO models, several researchers (Rijnaarts et al. 1999; Wang et al. 2011) reported that the attachment of *P. putida* was affected by the hydrophobic interaction and steric interaction. In order to compare the result of classical DLVO model with that of the extended DLVO model, Born interaction (V_Born_) was also adopted. The total interaction energy profile was obtained by the superposition of the energies of DLVO and Born interactions.

6 $$V_{Tot} = V_{EDL} + V_{VdW} + V_{Born}$$

The Born interaction energy was estimated by the following equation (Hahn et al. 2004).

7 $$V_{Born} = - \frac{{A\sigma_{B}^{6} }}{7560}\left[ {\frac{{8a_{p} + h}}{{(2a_{p} + h)^{2} }} + \frac{{6a_{p} - h}}{{h^{7} }}} \right]$$

where σ_B_ is Born collision diameter. A typical experimentally derived value for σ_B_ is 0.5 nm (Hahn et al. 2004). To check the effect of the Born interaction on the bacterial behavior, surface interaction energy was also calculated by extended DLVO models including Born interaction (Eq. 7). All parameters used for calculation of interaction energy are summarized in Additional file 1: Table S1.

## Results

### Effect of leaching solution on bacterial BTC

**Fig. 3:**
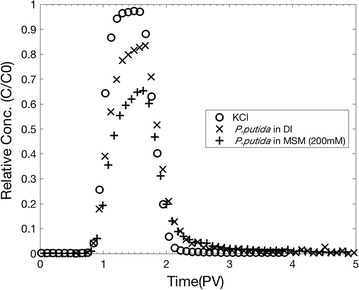
Breakthrough curves of KCl and *Pseudomonas putida* for leaching with deionized water and MSM (200 mM) (C_1_)

Figure 3 shows the breakthrough curves (BTCs) of KCl (Exp. A) and bacteria for two different leaching solutions of deionized water (Exp. B) and 200 mM MSM (Exp. C_1_). The peak concentrations and mass recoveries are presented in Table 3. The KCl BTC shows a symmetrical curve with nearly 100% elution without tailing, indicating that KCl behaved as a conservative tracer. The peak arrival times of bacteria nearly coincided with that of KCl although the peak concentrations of bacteria were lower than that of KCl. The lower peak of bacterial BTC of Exp. B indicates that physical factor such as deposition is responsible for the bacterial attachment to the sand surface since deionized water exhibited a null ionic strength. The physical deposition might result from the physical straining and the surface roughness. The much lower peak of bacteria BTC of Exp. C_1_ shows that physicochemical factor such as ionic strength of leaching solution influences the bacterial attachment. The increased attachment by increasing ionic strength is due to the increasing van der Waals attractive forces. Both bacterial BTCs have similar tailings. This may imply that the bacterial detachment occurred even under physicochemically stable condition. The only possible cause for this type of detachment might be physical interaction such as hydrodynamic shear. Other researchers also reported the increased deposition and the lower mass recovery at higher ionic strength. Deposition of *Escherichia coli* (Redman et al. 2004) and *Pseudomonas fluorescens* (Chen and Zhu 2004) increased for the increase in the ionic strength of leaching solution, and that the mass recovery of *Pseudomonas* sp. strain KL2 decreased from 57 to 44% during transport through 30 cm long quartz sand column when deionized water was switched to leaching solution of 10 mM NaCl (Gannon et al. 1991).

**Table 3:** Peak concentration, mass recovery (MR) and mass loss (ML) of BTCs of Exp. A, B, C

Exp.	A	B	C_1_	C_2_	C_3_
C_Peak_^a^	0.97	0.83	0.65	0.68	0.66
MR	0.99	0.92	0.72	0.80	0.87
ML	0.01	0.07	0.27	0.20	0.12

^a^ Peak concentration normalized to input concentration

### Effect of ionic strength on bacterial BTC

**Fig. 4:**
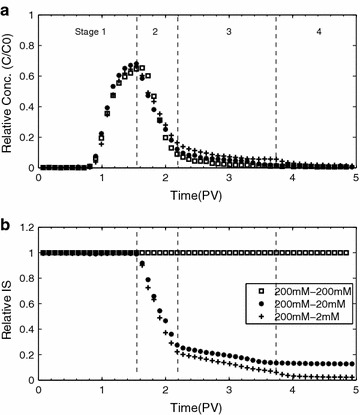
**a** Breakthrough curves of *Pseudomonas putida* and **b** ionic strength curves of effluent for Exp. C which used MSM (200 mM), tenfold (20 mM) and 100-fold (2 mM) diluted MSM as leaching solution after bacterial pulse

The effect of ionic strength of leaching solution after pulse on bacterial transport is presented in Fig. 4a. Substantial differences between bacterial BTCs were observed in the tailings although the peaks were nearly identical. Bacterial BTCs of Exp. C_2_ and C_3_ showed the increased tailings as the ionic strength of the effluent was lowered. The bacterial BTCs could be separated into four stages depending on the ionic strength of the effluent (Fig. 4b). In the first stage (0–1.55 PV), the ionic strengths remained at 200 mM while bacterial BTCs showed an identical rising limb and peak. During the second stage, the ionic strengths of Exp. C_2_ and Exp. C_3_ gradually decreased with time due to the dilution of pulse with leaching solutions of the lower ionic strengths. Despite the large discrepancy between the ionic strength of Exp. C_1_ and those of others, the falling limbs of the bacterial BTCs of Exp. C_2_ and C_3_ were almost identical with that of Exp. C_1_. In the third stage (2.2–3.8 PV), the bacterial BTCs showed different degree of the tailings. In the final stage, the ionic strengths reached to a steady-state condition with disappearance of the tailings.

## Discussion

### Bacterial detachment and DLVO theory

In the previous section, the bacterial concentrations in the tailing part were found to increase as the ionic strength was lowered. It is, however, questionable to predict the cause of the increased bacterial mass as a result of decreased ionic strength. Redman et al. (2004) reported that the increased bacterial mass was due to bacteria detachment upon the application of lower ionic strength. They found that bacterial attachment and detachment are partially reversible depending on the ionic strength of leaching solution.

**Fig. 5:**
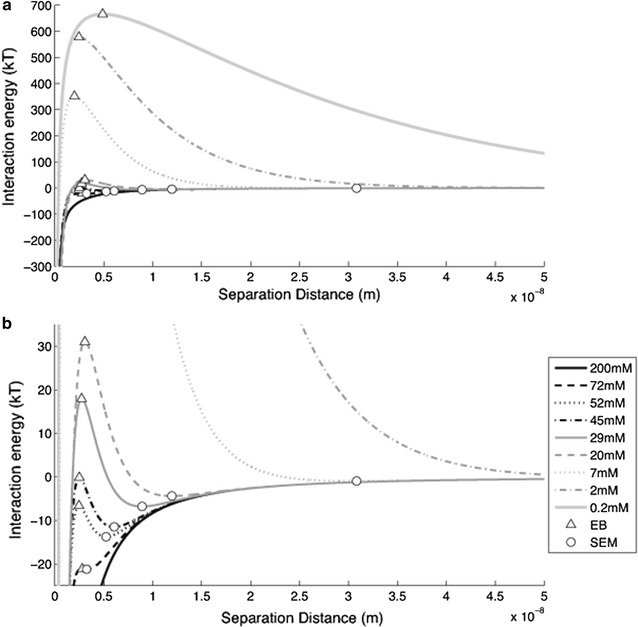
**a** Interaction energy as a function of separation distance and ionic strength calculated by Eq. (2). Identical data are replotted in **b** to highlight the energy barrier (EB) and secondary energy minimum (SEM)

In this study, we also investigated whether the bacterial attachment and detachment can be explained by DLVO or extended DLVO theory. The interaction energy profiles for various ionic strengths based on DLVO theory obtained using Eq. (2) are shown in Fig. 5. Detailed information on the height of energy barrier (V_EB_) and the depth of secondary energy minimum (V_SE_) at the separation distance is also given in Additional file 1: Table S2. Since the interaction energy profiles showed different curves depending on the ionic strength of solution, the ionic strengths were divided into the three different ranges according to the occurrence of secondary energy minimum. In the range of high ionic strength (IS > 72 mM), the profile has a primary energy minimum without energy barrier. Therefore bacteria can approach to the sand surface due to the attractive force, and thus a large amount of bacteria can be retained in the primary energy minimum. In the intermediate rage (IS = 3.8–72 mM), the profiles show secondary energy minima and energy barriers. The energy profile of 72 mM has a deep secondary energy minimum with the smallest energy barrier. The energy barrier becomes higher while the secondary energy minimum becomes negligible as ionic strength decreases. In this condition, the bacteria are dragged farther from the sand surfaces by the repulsive forces and retained around the secondary energy minimum. In the low range of ionic strength (IS < 3.8 mM), the interaction energy profiles have neither primary nor secondary energy minimum. This means that bacteria in solution experience only a repulsive force, and thus they cannot approach to the sand surface.

**Fig. 6:**
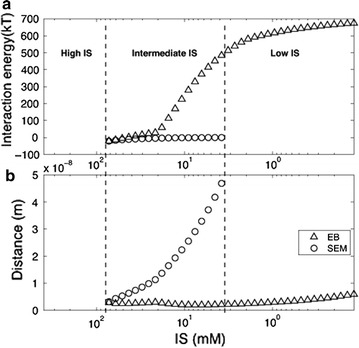
Changes in **a** interaction energy and **b** separation distance of energy barrier (EB) and secondary energy minimum (SEM) shown in the interaction energy profiles obtained from the simulation for wide range of ionic strength

Decrease of ionic strength from high to intermediate range makes the bacteria in the primary energy minimum separated by the energy barrier, and energy barrier develops and results in the formation of secondary energy minimum. Thus some part of the bacteria attached by the primary energy minimum is separated and retained by the secondary energy minimum. For further decreasing in the ionic strength (IS < 20 mM), the cells are gradually detached due to the increase in the energy barrier (Fig. 6a), and the secondary energy minimum migrates farther from the surface with the lower depth (Fig. 6b). Despite of the formation of secondary energy minimum, the bacteria deposited in the primary energy minimum can be retained stably and contributes to the irreversible attachment. This indicates that the reversibility of the bacterial attachment under physicochemical perturbation is depending on the depth of the secondary energy minimum and the separation distance, which are all affected by the ionic strength of leaching solution.

The interaction energy calculated could be related to the effect of ionic strength on the bacterial BTCs, which were shown in Fig. 4. The peak attenuation of bacterial BTCs in the stage 1 can be explained by bacterial attachment onto the primary energy minimum developed in the high range (200 mM) of ionic strength. Since sufficiently high concentration of bacteria was supplied, a large amount of cells is attached in the sand surface. Identical BTCs observed in the stage 2 can be explained by the fact that the range of ionic strength in pore water is too high to experience energy barrier or secondary energy minimum. In this range, the attached bacteria are not detached. In the stage 3, the tailings of the BTCs of Exp. C_2_ and C_3_ appeared. This is attributed to the development of the secondary energy minimum. In this condition, some part of the bacteria attached by the primary energy minimum was detached and thus contributes to the reversible attachment. In the stage 4, detachment vanishes as the secondary energy minimum disappears and accordingly almost no difference in the BTCs was found.

### Quantification of bacteria attachment and detachment

**Fig. 7:**
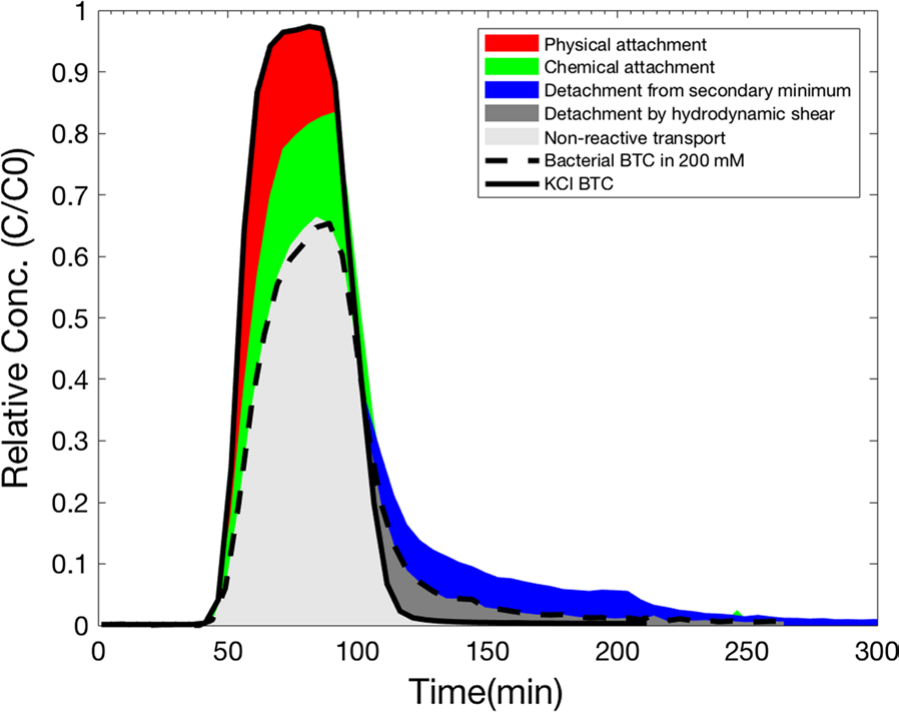
Quantification of bacterial attachment and detachment based on the KCl and bacterial BTC curves obtained from various leaching solutions

The BTCs with various bacterial peaks and mass recoveries (MR) obtained from the imposition of various ionic strengths are shown in Fig. 7. Mass changes due to the physical and physicochemical factors can be estimated the BTCs of Exp. B, C_1_ and C_3_ as a reference of KCl BTC (Exp. A) since the latter showed almost no mass change. The mass recovery of bacterial BTC of Exp. B consists of two different parts: (a) peak part with a mass reduction and (b) tailing part with a mass gain. The mass reduction at peak part is due to the physical attachment such as deposition. The mass gain at tailing part is due to the detachment of loosely held cells by the hydrodynamic shear (Bergendahl and Grasso 2003) since no physicochemical factors are involved during the pulse injection. The net mass loss of Exp. B (*MR*_*A*_ − *MR*_*B*_) is therefore physical attachment (*A*_*ph*_) minus hydrodynamic detachment (*D*_*h*_) and can be expressed as a fraction of irreversible attachment by physical deposition (*A*_*irr*_).

8 $$MR_{A} - MR_{B} = A_{ph} - D_{h} = A_{ph,irr}$$

where $D_{h,B }$ is the tailing part of Exp. B (D_h_ = 5%) gained by hydrodynamic shear implying the reversible fraction of physical attachment (*D*_*h*_ = *A*_*ph*,*rev*_). And the irreversible physical deposition is about 7% since the difference between mass recoveries of the BTCs of Exps. A and B is about 7% (Table 3). So the fraction of attached cells by the physical deposition process can be estimated as 12% by addition of reversible and irreversible fractions. Exps. C_1_, C_2_ and C_3_ showed similar reduction in peak part. The mass loss at peaks of Exps. C is due to the imposition of high ionic strength and thus mainly physicochemical attachment. The fraction of mass loss by chemical attachment (A_ch, C_) in high ionic strength condition can be estimated by using the mass recoveries of the BTCs of Exp. B and C_1_ due to their similar tailing part by assuming similar fraction of physical attachment.

9 $$A_{ch,C} = MR_{B} - MR_{C1}$$

The physicochemical attachment (A_ch, C_ = 20%) of Exps. C can be divided into irreversible and reversible processes so that $A_{ch,C} = A_{ch,irr} + A_{ch, rev}$. The reversible physicochemical attachment ($A_{ch, rev} = 15\%$) can be estimated from the tailing of BTC difference between Exp. C_1_ and C_3_.

10 $$A_{ch, rev} = MR_{C3} - MR_{C1}$$

And thus, the fraction of irreversible physicochemical attachment can be estimated as *A*_*ch*,*irr*_ = 5%. So the physicochemical attachment consisted of 3/4 and 1/4 of reversible and irreversible fraction respectively. In summary, in the high ionic strength condition, about 12 and 20% of the bacterial attachment occurred during transport by physical deposition and physicochemical attachment respectively; where among them, 5 and 15% of cells were attached reversibly respectively.

## Additional file

**Additional file 1: Table S1.** Parameters used for calculation of interaction energy between *P. putida* and quartz sand surface. **Table S2.** Zeta potential of quartz sand and *P. putida* used for calculation of energy barrier and secondary energy minimum for various ionic strengths.

## Abbreviations

MSM: mineral salt medium
MR: mass recovery
BTC: breakthrough curve
