# Supplementary material for: Quantifying bacterial attachment and detachment using leaching solutions of various ionic strengths after bacterial pulse
Source: AMB Express. 2017 Feb 14;7:38. doi: 10.1186/s13568-017-0340-2 (PMC5309194; doi:10.1186/s13568-017-0340-2)
Supplement: Supplementary file 1 — Additional file 1: Table S1. Parameters used for calculation of interaction energy between P. putida and quartz sand surface. Table S2. Zeta potential of quartz sand and P. putida used for calculation of energy barrier and secondary energy minimum for various ionic strengths. [file 13568_2017_340_MOESM1_ESM.docx]

**Table S1.** Parameters used for calculation of interaction energy between *P.putida* and quartz sand surface

| Parameter | Definition (unit) | Value | Remark |
| --- | --- | --- | --- |
| H | Separation distance (m) |  |  |
| ε_0_ | Vacuum permittivity (C^2^/N/m^2^) | 8.85e-12 |  |
| ε_r_ | Relative dielectric permittivity of water (-) | 80.1 |  |
| a_p_ | Equivalent spherical radius of the bacteria (m) | 1.5163e-6 |  |
| ψ_p_ | Surface potentials of the bacterial cell (V) |  | Table 5 |
| ψ_c_ | Surface potentials of the sand surface (V) |  | Table 5 |
| Κ | Inverse of Debye length (1/m) |  | Table 5 |
| I | Ionic strength (mol/L) |  | Table 5 |
| E | Electron charge (C) | 1.602e-19 |  |
| N_A_ | Avogadro’s number (molecules/mol) | 6.02e23 |  |
| T | Temperature (K) | 298.15 |  |
| K | Boltzmann’s constant (J/K) | 1.381e-23 |  |
| A | Hamaker constant (J) | 6.5e-21 |  |
| λ | Characteristic wavelength of the dielectric (nm) | 100 |  |

**Table S2.** Zeta potential of quartz sand and *P. putida* used for calculation of energy barrier and secondary energy minimum for various ionic strengths

| IS (mM) | Zeta  Potential (mV) | | Inverse  Debye  length  (1/m) | Energy barrier | | Secondary minimum | |  |
| --- | --- | --- | --- | --- | --- | --- | --- | --- |
|  | Sand | P.p |  | Height (kT) | Distance (nm) | Depth (kT) | Distance (nm) |  |
| 200.0 | -18.1 | -7.1 | 1.46e+09 | - | - | - | - |  |
| 72.0 | -32.0^*^ | -7.3^*^ | 8.73e+08 | -21.15 | 2.80 | -21.21 | 3.25 |  |
| 52.0 | -34.2^*^ | -7.3^*^ | 7.42e+08 | -6.62 | 2.47 | -13.74 | 5.24 |  |
| 45.0 | -35.0^*^ | -7.3^*^ | 6.90e+08 | -0.15 | 2.50 | -11.50 | 6.06 |  |
| 29.0 | -36.7^*^ | -7.4^*^ | 5.54e+08 | 17.93 | 2.74 | -6.81 | 8.91 |  |
| 20.0 | -37.7 | -7.4 | 4.60e+08 | 31.01 | 3.07 | -4.39 | 11.96 |  |
| 7.0 | -41.5^*^ | -17.0^*^ | 2.72e+08 | 350.93 | 2.00 | -0.95 | 30.83 |  |
| 2.0 | -43.0 | -20.7 | 1.46e+08 | 577.25 | 2.46 | - | - |  |
| 0.2 | -39.7 | -21.6 | 4.60e+07 | 665.49 | 4.89 | - | - |  |
| * Values obtained by interpolation. | | | | | | | | |
